# Within‐drug benefit‐risk evaluation of olanzapine long‐acting injection at one and two years of treatment

**DOI:** 10.1002/mpr.1443

**Published:** 2014-07-03

**Authors:** Holland C. Detke, John Lauriello, John Landry, David P. McDonnell

**Affiliations:** ^1^ Eli Lilly and Company Indianapolis IN USA; ^2^ University of Missouri Department of Psychiatry Columbia MO USA; ^3^ Eli Lilly Canada Danforth Ontario Canada

**Keywords:** olanzapine pamoate, long‐acting injection, antipsychotic, schizophrenia, benefit‐risk

## Abstract

We sought to evaluate the within‐drug benefit‐risk of olanzapine long‐acting injection (LAI) using both quantitative and qualitative methods. Subjects included 1192 adult patients with schizophrenia or schizoaffective disorder who participated in clinical trials with the opportunity for at least two years of continuous treatment with olanzapine LAI (45–405 mg every two to four weeks). Using the Benefit Risk Action Team (BRAT) framework, we evaluated frequency versus duration of benefits and risks commonly observed with atypical antipsychotics. We then used the Transparent Uniform Risk/Benefit Overview (TURBO) method, which weighs the drug's two most medically serious and/or frequent adverse events versus its primary benefit (effectiveness) and an ancillary benefit. The most frequent events among all patients were remaining free of relapse (91.4% for an average of 306 days at one year, 88.4% for 546 days at two years) and symptomatic remission (81.7% for an average of 239 days at one year, 84.1% for 438 days at two years). One‐ and two‐year incidence of ≥7% weight gain was 33.3% and 41.7%. Incidences for sexual dysfunction, hyperprolactinemia, and post‐injection delirium/sedation syndrome (PDSS) were <2%. TURBO ratings unanimously selected PDSS and weight gain as key risks and resulted in an average score in the acceptable benefit‐risk balance range. Copyright © 2014 John Wiley & Sons, Ltd.

## Introduction

Benefit‐risk assessment of a medication is a critical process not only at the regulatory level but also at the patient level. Just as regulators must weigh a drug's benefits and risks when deciding whether to approve its use in certain indications or populations, clinicians must also weigh these elements when deciding whether or not to prescribe the drug to a specific patient. Although many excellent quantitative and qualitative methods of benefit‐risk analysis have been described (see Guo *et al.*, 2010; Yuan *et al.*, 2011; CIOMS, 1998; EMA CHMP, 2008, for reviews), there is no standard or systematic method universally agreed upon for how best to conduct such an analysis. Moreover, given the multitude of variables and outcomes that can factor into the overall assessment, it can become very difficult to find a method that is not only sufficiently comprehensive in its breadth but also sufficiently clear and transparent so that it can be easily performed and understood. Additionally, the need for objectivity and standardization of methodology must also be balanced against the recognition that not all patients are the same and that some degree of subjectivity and flexibility is necessary to account for differing situations and clinical needs. Consequently, we sought to use a multi‐method approach in evaluating the benefit‐risk profile for olanzapine long‐acting injection (LAI).

Olanzapine LAI is a pamoate depot formulation of the antipsychotic olanzapine. Previous studies have established the efficacy and safety of olanzapine LAI in patients with schizophrenia treated for up to eight weeks of acute therapy (Lauriello *et al.*, 2008) or for up to 24 weeks of maintenance therapy (Kane *et al.*, 2010). The safety profile of olanzapine LAI has been shown to be consistent with that of oral olanzapine, with the exception of injection‐related adverse events, including post‐injection delirium/sedation syndrome (PDSS) (Kane *et al.*, 2010). Additional long‐term treatment studies have now also been completed (McDonnell *et al.*, 2011; Detke *et al.*, 2014), affording the opportunity to evaluate the benefit‐risk profile of this medication for longer periods of time that more closely mirror the reality of the long‐term nature of treatment for this chronic disease.

To evaluate benefit‐risk, we took a multi‐method approach that incorporated both quantitative and qualitative methods. For the first method, we identified key risks and benefits associated with atypical antipsychotics as well as injectable medications following the principles of the Benefit Risk Action Team (BRAT) framework (Coplan *et al.*, 2011). The BRAT framework is a structured approach to benefit‐risk assessment designed to assist with the simultaneous consideration of multiple efficacy and safety endpoints (Coplan *et al.*, 2011; Levitan *et al.*, 2011). Developed by the Pharmaceutical Research and Manufacturer's Association in consultation with regulatory agencies to increase transparency and consistency of benefit‐risk assessments during drug development and regulatory review, this framework provides a set of flexible processes and tools to guide selection, organization, understanding, and summarization of evidence relevant to benefit‐risk decisions. Within this framework, we then applied quantitative analyses to develop a unified visual presentation of the risks and benefits identified through the BRAT framework. Levitan (2011) also applied the BRAT framework and convincingly argued for the importance of finding a concise way to display multiple endpoints. While Levitan's specific choice of quantitative methods was based on the desire to display comparative, between‐drug data, our goal was to provide a within‐drug method of analysis – i.e. a method to weigh the benefits versus risks within a single drug rather than to compare profiles between drugs. Consequently, we chose to present the relative impact of multiple endpoints by plotting the frequency of an outcome versus the average time spent experiencing that outcome. This analysis provides a rough proxy for magnitude of impact of an event, while also placing the data for that event within the context of all the other events evaluated. This single, visual benefit‐risk profile can then be evaluated as a whole to help the viewer form an opinion as to whether the benefits outweigh the risks of the drug for that patient population as a whole or, specifically, for an individual patient for whom the drug is being considered.

For the second method, we selected the Transparent Uniform Risk/Benefit Overview (TURBO) method. This is a semi‐structured rating method that weighs subjective ratings of a drug's primary benefit (treatment effectiveness) and ancillary benefits versus a drug's most potentially medically serious and/or frequent adverse events. The TURBO method was developed by Dr Willem Avery and selected by the Council for International Organizations of Medical Sciences (CIOMS) for inclusion in their guidance document on risk‐benefit assessment (CIOMS, 1998). CIOMS is an international non‐governmental organization founded in 1949 to serve the World Health Organization (WHO) that provides consultation and guidance on topics such as bioethics and drug development.

## Method 1 – BRAT framework and analysis of frequency versus duration

The BRAT framework (Coplan *et al.*, 2011) consists of six steps: defining decision context, identifying outcomes, identifying data sources, customizing the framework (i.e. specifying the analyses to be used based on the constraints of the data), assessing outcome importance, and displaying and interpreting the key benefit‐risk metrics. We present our quantitative olanzapine LAI analysis method using this framework.

### Step 1. Define the decision context

We chose to evaluate the within‐drug benefit‐risk profile of olanzapine LAI in patients with schizophrenia treated with this depot medication for up to one year and for up to two years.

### Step 2. Identify outcomes

**Figure 1:**
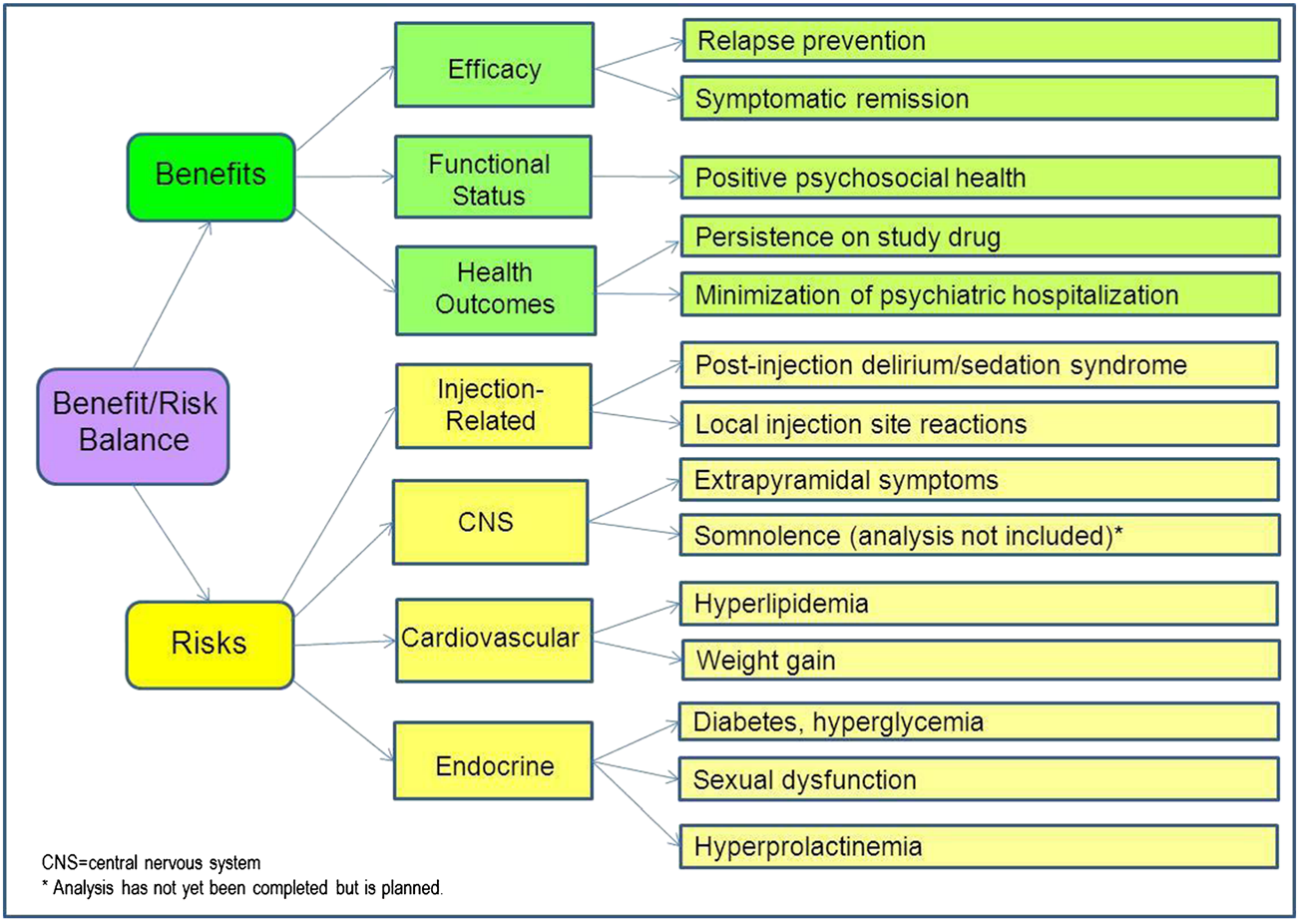
BRAT framework value tree.

Markowitz *et al.* (2011) identified key benefits and risks typically associated with atypical antipsychotics based on a review of the scientific literature and package inserts and in consultation with clinical experts. These benefits and risks fell into the categories of clinical response, functional status, health outcomes, central nervous system, cardiovascular, and endocrine. We used these same general categories but also added a category for injection‐related events to account for the route of delivery of the LAI medication. We then modified the specific outcomes associated with each category and also renamed “clinical response” to “efficacy” in order to reflect the specific outcome measures that were used in the olanzapine LAI trials and to reflect the long‐term nature of the data, which was geared toward maintenance treatment and relapse prevention as opposed to acute treatment and response. The resulting value tree is presented in Figure 1. Definitions of each outcome event are presented in Table 1.

**Table 1:** Definitions of outcome events

Outcome	Measures and Definition
*Benefits*	
Relapse prevention	Relapse was defined as one of three events: (1) hospitalization for symptoms related to worsening of psychosis or for suicidal or aggressive behavior, (2) specific changes in PANSS^a^ or BPRS^a^ total scores^b^ plus CGI‐S > 4, or (3) discontinuation from the study because of worsening of psychosis or for suicidal or aggressive behavior
Symptomatic remission	Score ≤ 3 (“mild” or better) on each of eight key PANSS symptom items^a^
Positive psychosocial health	Short Form Health Survey (SF‐36)^a^ Mental Composite Score ≥ 45
Persistence on study drug	Total days on study drug during each time period
Minimization of psychiatric hospitalization	Psychiatric hospitalization was defined as hospitalization due to “Primary study condition”
*Risks*	
Post‐injection delirium/sedation syndrome (PDSS)	All PDSS events (PDSS Algorithm^a^ three symptoms of olanzapine overdose or patient unconscious/stuporous; not due to dose increase, concomitant medications, or general medical condition)
Local injection‐site reactions	All injection‐site‐related treatment‐emergent adverse events (TEAEs)^c^
Extrapyramidal symptoms (EPS)	Any treatment‐emergent parkinsonism (Simpson–Angus^a^ total score > 3 at any time if baseline ≤ 3), akathisia (Barnes Akathisia Scale^a^ score of ≥2 at any time with score < 2 at baseline), or dyskinesia (Abnormal Involuntary Movement Scale^a^ single‐item score ≥ 3 or at least two item scores ≥ 2 at any time if all single‐item baseline scores < 3 and at least six item scores < 2 at baseline)
Hyperlipidemia	Treatment‐emergent abnormal lipids were defined as a baseline of < 3.390 mmol/l triglycerides and < 6.216 mmol/l cholesterol at baseline to ≥ 3.390 mmol/l triglycerides or ≥ 6.216 mmol/l total cholesterol at any time post baseline
Clinically significant weight gain	Weight gain ≥ 7% of baseline
Diabetes, hyperglycemia	All diabetes or hyperglycemia‐related adverse events^d^ and/or start of glucose‐lowering medications^e^
Sexual dysfunction	All sexual‐related TEAEs^f^
Hyperprolactinemia	All prolactin‐related TEAEs^g^

Abbreviations: BPRS = Brief Psychiatric Rating Scale; PANSS = Positive and Negative Syndrome Scale; TEAEs = spontaneously reported treatment‐emergent adverse events.

a PANSS total scores (Kay *et al.*, 1987); BPRS total scores (Overall and Gorham, 1962); eight key PANSS symptom items (Andreasen *et al.,* 2005); SF‐36 (Ware *et al.*, 1993); PDSS Algorithm (Detke *et al.*, 2010); Simpson–Angus total score (Simpson and Angus, 1970); Barnes Akathisia Scale (Barnes, 1989); Abnormal Involuntary Movement Scale (Guy 1976).

b If PANSS was collected, either: (a) an increase of 25% from baseline if PANSS total baseline > 40, or (b) an increase ≥ 10 points if PANSS total baseline ≤ 40. If PANSS not collected, then either (a) a BPRS total score increase of 25% from baseline if BPRS total baseline > 24, or (b) an increase ≥ 6 points if BPRS total baseline ≤ 24.

c Injection‐related events (observed): erythema induratum or “injection site” nodule, induration, hematoma, pruritis, erythema, pain, reaction, mass, swelling, abscess, warmth, paresthesia, extravasation, hemorrhage, anesthesia, discoloration, inflammation, irritation oedema, rash.

d Diabetes‐related events (observed): blood glucose increased, hyperglycemia, diabetes mellitus, type 2 diabetes mellitus, glucose tolerance impaired, diabetic ketoacidosis, glucose tolerance decreased, glycosylated hemoglobin increased, insulin resistance.

e Glucose‐lowering medications (observed): acarbose, avandamet, benfluorex, glibenclamide, glibomet, gliclazide, glimeperide/metformin, glimepiride, glipizide, metformin, metformin/glibenclamide, metformin/rosiglitazone, pioglitazone, repaglinide, rosiglitazone, sitagliptin, insulin.

f Sexual‐related events (observed): ejaculation delayed, erectile dysfunction, libido decreased, priapism, sexual dysfunction.

g Prolactin‐related events (observed): hyperprolactinemia, breast discharge, amenorrhea, menstrual disorder.

### Step 3. Identify data sources

To evaluate benefit‐risk in the context of long‐term treatment, we used data from all patients in the integrated olanzapine LAI clinical trials database that had the opportunity to receive up to at least two years of continuous treatment with olanzapine LAI. Thus, patient data came predominantly from two clinical trials: a six‐year, open‐label, extension study, F1D‐MC‐HGKB (*n* = 931; McDonnell *et al.*, 2011), and a two‐year, open‐label, randomized study, F1D‐MC‐HGLQ (*n* = 264; Detke *et al.*, 2014). Patients from the six‐year study had participated in one of three previous feeder studies: an eight‐week acute study F1D‐MC‐HGJZ (*n* = 199; Lauriello *et al.*, 2008), a 24‐week relapse prevention study F1D‐MC‐HGKA (*n* = 642; Kane *et al.*, 2010), or a four‐week, single‐injection, pharmacokinetic study, F1D‐EW‐LOBS (*n* = 90). Patients in the six‐year study were 18 to 76 years of age, diagnosed with schizophrenia (*n* = 909) or schizoaffective disorder (*n* = 22) using the Diagnostic and Statistical Manual of Mental Disorders, 4th Edition, Text Revision (DSM‐IV‐TR). Clinical status at time of study entry was dependent on the nature of the feeder study, with most patients being clinically stable, except for those from the eight‐week acute study who were still in the process of stabilizing. Dosing in the six‐year study was initiated at 210 mg/two weeks for the first two weeks and was flexible thereafter in the range of 45 to 450 mg every two to four weeks. Patients in the two‐year study were 18 to 65 years of age, diagnosed with DSM‐IV‐TR schizophrenia, and were clinically stable at the time of entry into the study. Dosing in the two‐year study was initiated at 405 mg/four weeks for the first eight weeks and was flexible thereafter in the range of 150 to 405 mg/four weeks. Patients were eligible for inclusion in the analyses if they had at least one injection of olanzapine LAI.

### Step 4. Customize the framework

As described earlier, we customized the framework by adapting the benefit‐risk value tree to align with the specific outcomes that were assessed across both of the long‐term trials and to include events unique to an injectable medication.

### Step 5. Assess outcome importance

We chose to use a purely quantitative method to examine outcomes by presenting the frequency of identified outcome events versus the mean time spent experiencing those events. Baseline for the analysis was time of first therapeutic olanzapine LAI dose (150 to 300 mg/two weeks or 150 to 405 mg/four weeks); thus, baseline occurred at first injection in the feeder study for patients assigned to a therapeutic dose in the feeder. To be included in scale‐ and laboratory‐based assessments, patients had to have both a baseline and at least one post‐baseline assessment. Rates and mean times with event reflected data for all patients rather than patients with the event, unless otherwise specified. For spontaneous adverse events, time with event was calculated counting from start to stop date or study completion date. For scale‐ and laboratory‐based events, time with event was based on time between the visit at which the patient first met criteria for the event and the visit at which criteria were no longer met or at which time the patient completed the study.

### Step 6. Display and interpret key benefit‐risk metrics

Results are presented at one‐ and two‐year cutoffs in both graphic and tabular form.

## Results 1 – BRAT framework and analysis of frequency versus duration

### Patient characteristics

Table 2 summarizes the demographics and disease state of the patients included in the analysis. Patients were on average 40 years of age, and predominately male (66.5%), White (66.1%), and with a diagnosis of schizophrenia (97.6%). Patients were predominantly clinically stable at baseline (Positive and Negative Syndrome Scale [PANSS] total score mean: 61.7 [standard deviation (SD) = 22.6]), with the exception of those patients coming from the acute feeder study, whose baseline PANSS total scores averaged approximately 100.

**Table 2:** Patient characteristics at analysis baseline

Characteristic	Olanzapine LAI (*N* = 1192)
Age in years, mean (SD)	39.7 (11.6)
Male (%)	66.5
Race (%)	
Caucasian	66.1
Hispanic	13.9
African	11.9
E. Asian	5.2
W. Asian	2.4
Native American	0.4
DSM‐IV‐TR Diagnosis (%)	
Schizophrenia	97.6
Schizoaffective disorder	2.4
PANSS total score,^a^ mean (SD)	61.7 (22.6)
CGI‐S score, mean (SD)	3.2 (1.1)

Abbreviations: CGI‐S = Clinical Global Impressions‐Severity; DSM‐IV‐TR = Diagnostic and Statistical Manual of Mental Disorders, Fourth Edition, Text Revision; LAI = long‐acting injection; PANSS = Positive and Negative Syndrome Scale; SD = standard deviation.

a *N* = 1108; Patients were predominantly clinically stable at baseline with the exception of those patients coming from the acute feeder study, whose baseline PANSS scores averaged approximately 100.

### Dosing

The most common dose regimens were 405 mg/four weeks and 300 mg/two weeks. Mean daily olanzapine dose equivalent for all patients in the analysis was 14.1 mg/day, SD = 3.6.

### Frequency versus duration

**Figure 2:**
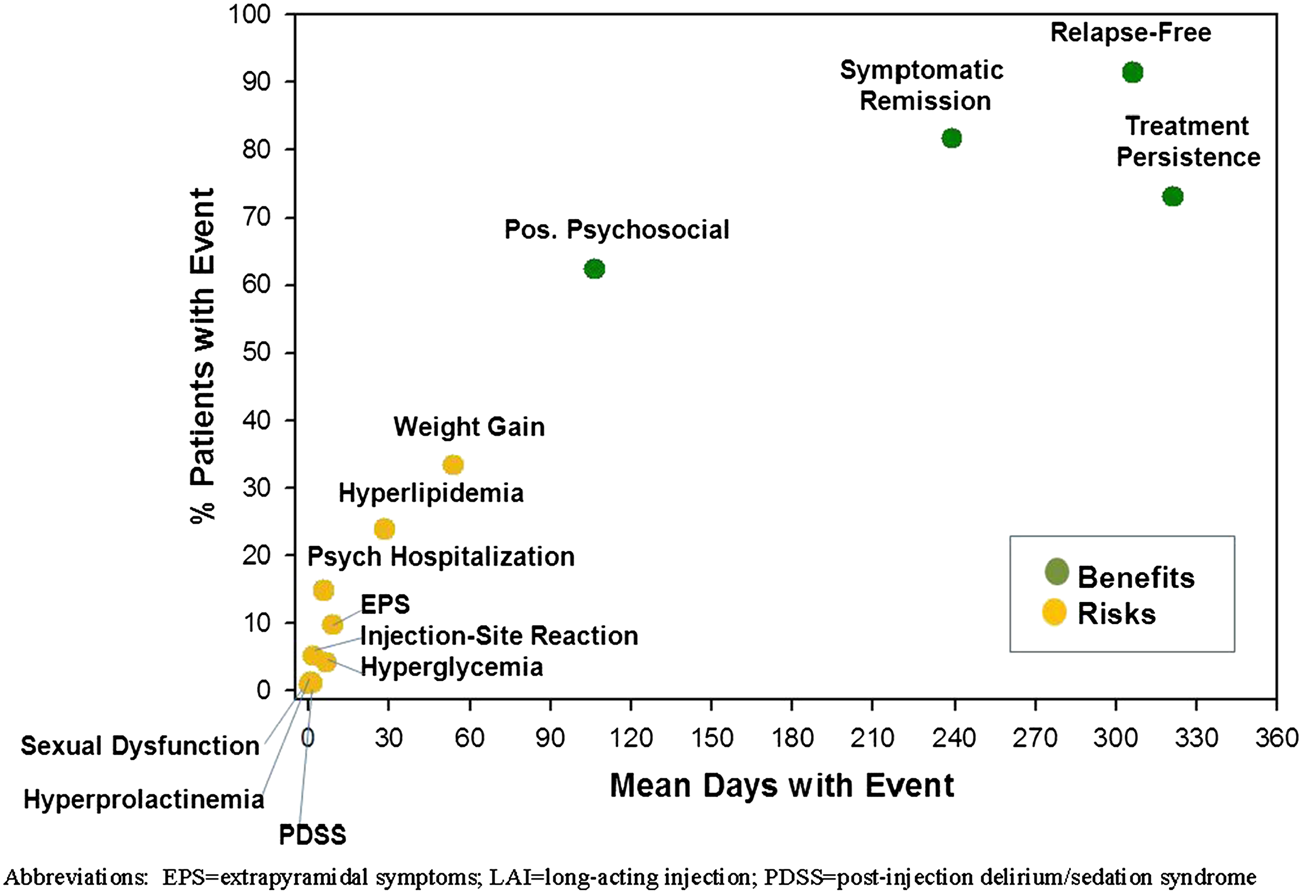
Frequency versus duration of key benefits and risks in patients treated with olanzapine LAI for up to one year.

**Figure 3:**
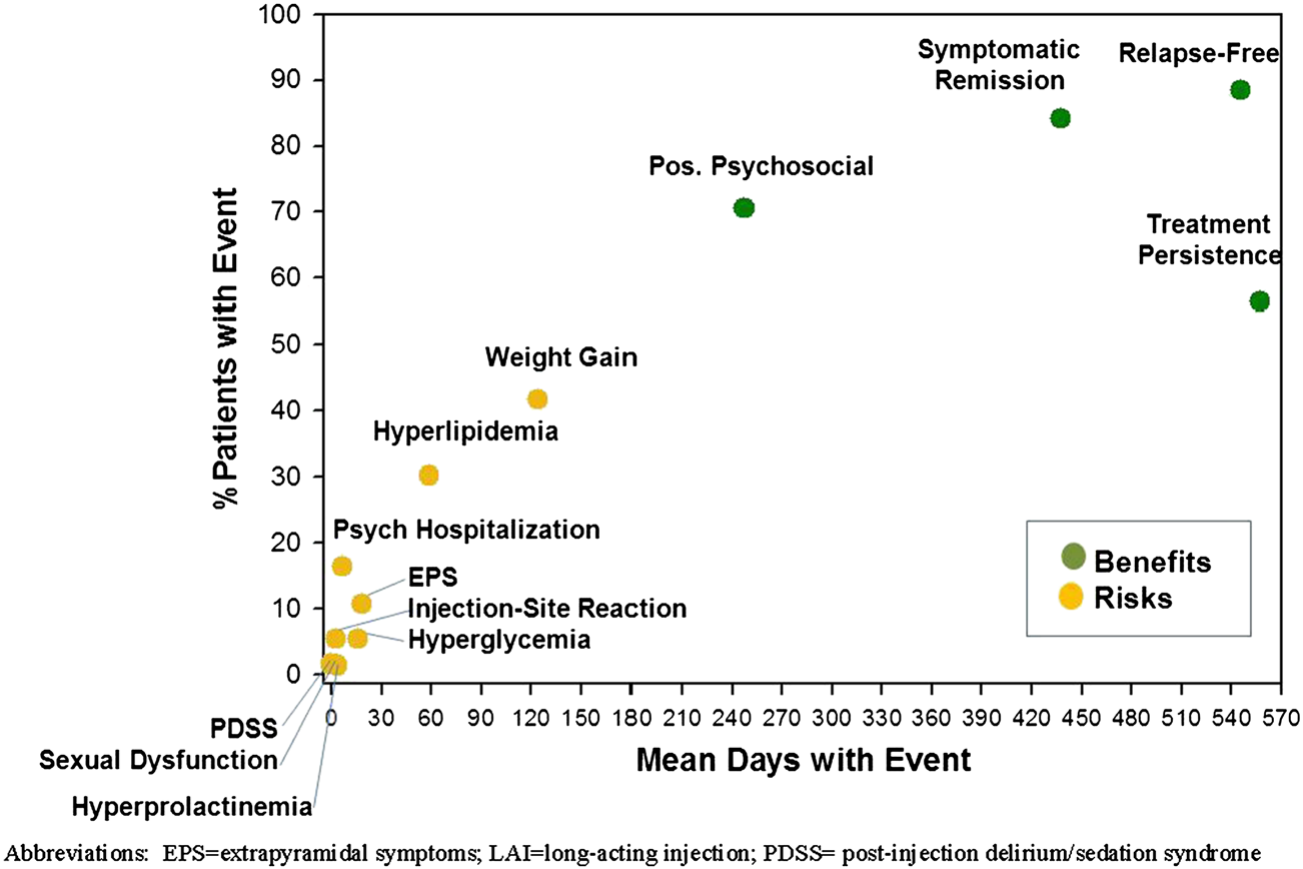
Frequency versus duration of key benefits and risks in patients treated with olanzapine LAI for up to two years.

Results at one year of treatment are presented in Table 3 and illustrated graphically in Figure 2; results at two years of treatment are presented in Table 4 and illustrated graphically in Figure 3. All benefits occurred more frequently and for longer durations than did any of the risks. The most frequent occurrence among all patients was remaining free of relapse (91.4% at one year and 88.4% at two years). Mean cumulative number of days without relapse was 306 (SD = 115) at one year and 546 (SD = 264) at two years. The next most frequent occurrence was symptomatic remission (a score of ≤3 on eight key PANSS items) at any assessment point (81.7% at one year and 84.1% at two years). Mean cumulative number of days meeting symptomatic remission criteria was 239 (SD = 156) at one year and 438 (SD = 301) at two years.

**Table 3:** Frequency and duration of outcome events after olanzapine LAI treatment for up to one year

Event	*N*	*n*	Percent	Mean days with event (SD)	
				For all patients	For patients with event
Relapse‐free	1192	1089	91.4	306.3 (114.6)	—
Symptomatic remission	1151	940	81.7	238.9 (156.1)	292.5 (118.9)
Persistence on study drug	1192	871	73.1	321.3 (110.7)	—
Positive psychosocial health	1126	701	62.3	106.6 (110.9)	171.2 (93.3)
Weight gain ≥ 7%	1185	395	33.3	54.0 (99.2)	162.1 (109.7)
Hyperlipidemia	852	203	23.8	28.5 (65.2)	119.4 (83.5)
Psychiatric hospitalization	1192	175	14.7	5.6 (24.2)	38.2 (52.4)
EPS (any)	959	92	9.6	9.0 (38.6)	94.1 (87.2)
‐ Parkinsonism	1009	63	6.2	5.8 (30.2)	93.0 (81.1)
‐ Akathisia	1075	37	3.4	2.3 (16.2)	67.6 (57.5)
‐ Dyskinesia	1093	28	2.6	3.1 (25.5)	120.1 (108.7)
Local injection‐site reactions	1192	60	5.0	1.8 (19.3)	35.3 (79.5)
Diabetes, hyperglycemia	1192	49	4.1	6.6 (42.0)	160.2 (136.2)
Sexual dysfunction	1192	13	1.1	0.8 (13.2)	77.5 (104.4)
Hyperprolactinemia	1192	12	1.0	1.2 (16.2)	118.0 (115.9)
PDSS	1192	9	0.8	0.0 (0.2)	2.2 (1.2)

Abbreviations: EPS = extrapyramidal symptoms; LAI = long‐acting injection; PDSS = post‐injection delirium/sedation syndrome; SD = standard deviation.

**Table 4:** Frequency and duration of outcome events after olanzapine LAI treatment for up to two years

Event	*N*	*n*	Percent	Mean days with event (SD)	
				For all patients	For patients with event
Relapse‐free	1192	1054	88.4	545.6 (264.2)	‐
Symptomatic remission	1151	968	84.1	437.6 (300.9)	520.4 (254.1)
Positive psychosocial health	1128	795	70.5	247.1 (241.9)	350.6 (216.1)
Persistence on study drug	1192	672	56.4	557.3 (259.0)	—
Weight Gain ≥7%	1185	494	41.7	123.7 (210.0)	296.6 (233.5)
Hyperlipidemia	858	259	30.2	58.6 (124.2)	194.2 (157.4)
Psychiatric hospitalization	1192	194	16.3	6.5 (34.0)	39.9 (76.1)
EPS (any)	964	102	10.6	17.9 (75.6)	169.5 (168.9)
‐ Parkinsonism	1014	70	6.9	12.5 (60.9)	180.8 (153.7)
‐ Akathisia	1080	43	4.0	4.8 (35.4)	121.4 (132.7)
‐ Dyskinesia	1098	33	3.0	6.1 (48.1)	201.7 (196.9)
Local injection‐site reactions	1192	64	5.4	2.7 (35.6)	50.8 (146.7)
Diabetes, hyperglycemia	1192	64	5.4	15.6 (90.6)	290.3 (272.2)
PDSS	1192	18	1.5	0.0 (0.3)	2.4 (1.4)
Sexual dysfunction	1192	17	1.4	1.7 (21.3)	119.4 (137.1)
Hyperprolactinemia	1192	16	1.3	3.2 (39.3)	236.1 (253.1)

Abbreviations: EPS = extrapyramidal symptoms; LAI = long‐acting injection; PDSS = post‐injection delirium/sedation syndrome; SD = standard deviation.

The most frequent risks were weight gain and hyperlipidemia. Incidence of clinically significant weight gain (≥7% of body weight) was 33.3% at one year and 41.7% at two years. Mean number of days with clinically significant weight gain was 54 (SD = 99) at one year and 124 (SD = 210) at two years, although among those patients affected, mean number of days with the event was 162 (SD = 110) at one year and 297 (SD = 233) at two years. Incidence of hyperlipidemia was 23.8% at one year and 30.2% at two years. Mean number of days with hyperlipidemia was 29 (SD = 65) at one year and 59 (SD = 124) at two years, although among those patients affected, mean number of days with the event was 119 (SD = 83) at one year and 194 (SD = 157) at two years.

Less frequent risks were extrapyramidal symptoms (EPS), injection‐site reactions, and diabetes/hyperglycemia, and least frequent risks were sexual dysfunction, hyperprolactinemia, and PDSS. Per‐patient incidence of PDSS was 0.8% at one year and 1.5% at two years. The vast majority of patients did not experience a PDSS event (mean number of days with PDSS was zero at one year and zero at two years); for those who experienced an event (nine patients at one year, 18 patients at two years), mean number of days with PDSS was two at one year and two at two years.

## Method 2 – TURBO method

We also evaluated the benefit‐risk profile of olanzapine LAI using the subjective TURBO method. Each of five raters independently applied the TURBO criteria to subjectively weigh olanzapine LAI's two most potentially medically serious and/or frequent adverse events (as subjectively identified by that rater) versus its primary benefit (treatment effectiveness) and an ancillary benefit (also subjectively identified by that rater). Ratings were averaged across raters and placed on a *T*‐score grid ranging from one (worst balance) to four (restricted access or further research necessary) to seven (excellent balance).

### Step 1. Calculate the risk‐factor score

Based on their existing knowledge of olanzapine LAI, each of the five raters (the four authors plus one consultant) independently identified what they considered to be the two most important risk factors for the drug. The first risk factor (*R*_1_) was to represent what they considered to be the most medically serious adverse event for the drug. The second risk factor (*R*_2_) was to represent either the next most medically serious adverse event for the drug or its most frequent adverse event. The impact of each of these adverse events on the patient's health status and socioprofessional capabilities was then independently rated on the following scale under the assumption that the event was detected in a timely manner and was appropriately medically managed:

1 =: some hindrance, but not really incapacitating
2 =: temporarily/intermittently incapacitating
3 =: incapacitating, but not life‐threatening or shortening
4 =: life‐shortening, but not life‐threatening
5 =: life‐threatening

A final risk‐factor score was then based on the rating for *R*_1_ plus any “correction factor” points based on the impact rating for *R*_2_. If *R*_2_ was <4, then no additional correction factor was applied to *R*_1_. However, if *R*_2_ = 4, then a correction factor of +1 was applied to *R*_1_, and if *R*_2_ = 5, then a correction factor of +2 was applied to *R*_1_. For example, if “PDSS” was selected as *R*_1_ and was given a rating of two (temporarily incapacitating), and if “weight gain” was selected as *R*_2_ and given a rating of four (life‐shortening, but not threatening), then the final risk‐factor score would be three (i.e. two plus a correction factor of +1).

### Step 2. Calculate the benefit‐factor score

Based on their existing knowledge of olanzapine LAI, each of the five raters independently rated the treatment effectiveness of the drug using the following scale of impact on the patient's disease state (in this case, schizophrenia) and socioprofessional capabilities:

1 =: less hindering, but capabilities remain unchanged
2 =: less frequently incapacitating or incapability lasts shorter
3 =: less incapacitating, but no change in life expectancy
4 =: less life‐shortening
5 =: less immediately life‐threatening

Raters were instructed to assume that the drug was being used correctly. Raters then were asked to identify independently any relevant ancillary benefit to the patient beyond treatment effectiveness. If this ancillary benefit was medical in nature (such as an antipsychotic drug that treats EPS), then a correction factor of +2 was applied. If this ancillary benefit was practical in nature (such as a drug that requires less frequent dosing), then a correction factor of +1 was applied. For example, if the treatment effectiveness of olanzapine LAI was rated as a four (schizophrenia is less life‐shortening), and an ancillary benefit of “monthly dosing” is selected (a practical property), then the final benefit‐factor score would be five (i.e. four plus a correction factor of +1).

### Step 3. The TURBO diagram

Each of the raters' final benefit‐factor scores were then plotted versus their final risk‐factor scores on a *T*‐score grid that describes different levels of benefit‐risk balance. Benefit‐factor scores and risk‐factor scores were also averaged across the five raters, and the average benefit‐factor score and risk‐factor score were also plotted against each other. The *T*‐scores could range from one (worst benefit‐risk balance) to four (restricted use only or requiring further study) to seven (best benefit‐risk balance).

## Results 2 – TURBO method

**Figure 4:**
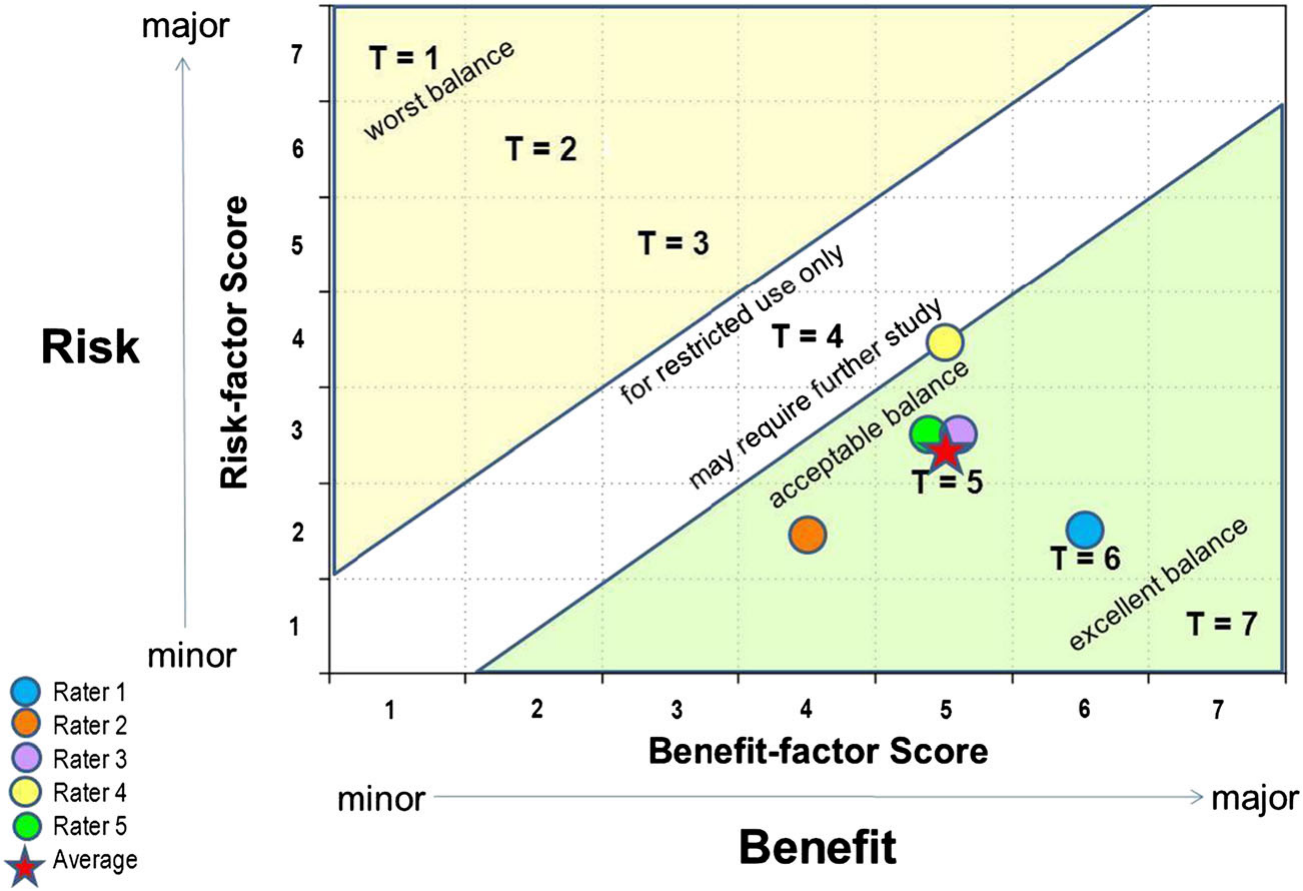
Placement of olanzapine LAI TURBO ratings on benefit‐risk *T*‐score grid.

For the TURBO analysis, raters unanimously selected PDSS and weight gain as key risks, although choice of ancillary benefit varied among raters (e.g. “infrequent dosing,” “improved compliance,” or “fewer relapses”) (Table 5). Mean benefit‐factor rating was five out of a possible seven. Mean risk‐factor rating was 2.8 out of a possible seven, yielding a mean benefit‐risk balance within the acceptable range (*T*‐score = 5), even when accounting for inter‐rater differences in subjective weightings. Placement of the olanzapine LAI TURBO ratings on the *T*‐score grid is provided in Figure 4.

**Table 5:** Olanzapine LAI TURBO ratings

Rater	Risk 1	Risk 1 rating	Risk 2	Correction factor from risk 2 rating	Primary benefit (effectiveness) rating	Ancillary benefit	Correction factor from ancillary benefit	Risk‐factor score	Benefit‐factor score
#1	PDSS	2	Weight gain	+0	4	Fewer relapses	+2	2	6
#2	PDSS	2	Weight gain	+0	3	Monthly injection	+1	2	4
#3	PDSS	2	Weight gain	+1	3	Fewer relapses	+2	3	5
#4	PDSS	3	Weight gain	+1	4	Identify non‐compliance	+1	4	5
#5	PDSS	2	Weight gain	+1	4	Infrequent dosing	+1	3	5
Average								2.8	5.0

Abbreviations: LAI = long‐acting injection; TURBO = Transparent Uniform Risk/Benefit Overview; PDSS = post‐injection delirium/sedation syndrome.

## Discussion

Benefit‐risk assessment continues to be an important but not well‐defined activity. Moreover, selection of methods may depend on the type of data that are available. In this analysis, we sought to evaluate the long‐term, within‐drug benefit‐risk profile of olanzapine LAI by assessing its benefits relative to its risks at one and two years of treatment. Using the BRAT framework, we developed a within‐drug method for presenting a unified picture of benefits versus risks by plotting frequency versus duration for key risks and benefits. We found that the large majority of patients treated with olanzapine LAI for up to one or two years of treatment experienced long periods of benefit, including extended relapse‐free periods and periods of symptomatic remission. For instance, at two years, 88% of patients remained free of relapse, and 84% met criteria for symptomatic remission. Risks were notably less frequent and of shorter duration, although potentially clinically significant weight gain and hyperlipidemia were the most frequent of these, occurring in 42% and 30% of patients respectively at two years. PDSS, which is a risk specific to the long‐acting formulation of olanzapine, was one of the least frequent risks, occurring in 1.5% of patients treated up to two years, with an average duration of zero days for all patients (or an average of two days for those patients who experienced the event). Using the TURBO method, we selected PDSS and weight gain as key risks, although the choice of ancillary benefit varied slightly among raters, depending on whether the primary advantage of a depot formulation was viewed as a medical benefit or a practical benefit. Comparison of the mean benefit and risk ratings across raters yielded a mean benefit‐risk balance within the acceptable range (*T*‐score = 5).

The specific analytic approaches used in this paper provide differing, although potentially complementary, methods of weighing benefits versus risks. Each method provides a unified view of the drug's key benefits and risks. The frequency‐versus‐duration analysis provides a straightforward approach to analyzing a large number of variables and results in a single graph that can easily be used to place benefits and risks in context with each other. The TURBO method also provides a simplified view of a complex set of variables by identifying the top two benefits and the top two risks. Depending on the two benefits and risks identified, different numerical values are ascribed to each, leading to a final value that illustrates either an overall benefit or an overall risk associated with treatment. Using the two methods together provides some degree of validation of the findings in each. For instance, the data‐driven approach of the first method provides support for the subjective results of the second method, and the single *T*‐score result of the second method provides additional subjective guidance for evaluating the relative balance of benefits and risks in the first method.

Nevertheless, it should be noted that both methods have strengths and limitations. A key strength of the BRAT framework has been its development within the pharmaceutical industry in consultation with regulators in order to provide a transparent and comprehensive analysis of benefits and risks. Within the BRAT framework, we selected a frequency‐versus‐duration analysis to provide a consistent and objective way to put specific long‐term benefits and risks into overall context while still allowing the viewer to make his/her own determination about relative importance of each. One limitation of this approach is that objective methods of assessment often still contain a subjective element based on the choice of events and outcome measures to assess. Moreover, interpretation may be limited by difficulty comparing subjective value of efficacy outcomes versus safety outcomes. An important methodological limitation in this particular analysis was that assessment of duration could be influenced by infrequency of certain assessment measures. For instance, in the six‐year study (HGKB), PANSS was only assessed every six months in the later years of study participation. Therefore, for some patients, symptomatic remission criteria may appear to have been met during a six‐month interval, but cannot be confirmed, as assessments did not occur periodically during that interval. Another limitation is that duration of an event could be limited by a patient's early discontinuation from the trial. Nevertheless, the relative durations across events in the present analyses do speak to the likelihood of an event being temporary versus of ongoing duration. For instance, the brief mean duration for PDSS events is indicative of the transient nature of this event, and the brief mean duration for hyperprolactinemia is indicative of the finding that elevations in prolactin during treatment with olanzapine LAI may be transient in some patients as well. In contrast, the longer durations for potentially clinically significant weight gain and hyperlipidemia may be more indicative of events that may be ongoing at the time of study discontinuation or completion.

One of the strengths of the TURBO model is that the application of the formula is very simple and can be used by anyone (for instance, a clinician, patient, or regulatory scientist) based on their knowledge of, and experience with, a product. Thus, the model can be used flexibly in different situations and using different raters, depending on the perspectives that are being sought. For instance, the patient perspective would be an important and valuable addition – certainly central to any personal decision‐making at the patient level but also potentially informative for higher level decisions, even at the regulatory level. Another strength is that this method produces a single unified *T*‐score that allows for the placement of the product on a benefit‐risk grid that can guide clinical usage. Limitations, however, include the fact that the method relies heavily on subjective ratings. Thus, any ratings must always be interpreted within the context of the source of the ratings, taking into consideration the rater's degree of knowledge and experience as well as unique perspectives, priorities, and biases in making the ratings. Another limitation is that the TURBO method only takes into consideration the two most medically serious and/or frequent adverse events for a medication as well as the primary and a single ancillary benefit, thus potentially ignoring other additional important information about a drug. It is also important to note that the TURBO model has not been validated, and anchors for the impact ratings were originally published as suggestions (CIOMS, 1998) and may not apply well to all disease states. Nevertheless, we found the method to be a useful and informative exercise.

The use of both methods together allows for some compensation for the limitations of each. Moreover, the ability to apply these techniques to the analysis of long‐term data that is less likely to be placebo‐ or comparator‐controlled potentially allows for a more relevant view of a drug's benefit‐risk profile for real world clinical practice. Many psychiatric illnesses are long‐term in nature, and these analytic approaches may be useful in assessing benefits and risks for such chronic disease states. Therefore, value can be gained in looking at the benefits and risks associated with long‐term treatment with only one medication. Ideally, similar methods could be applied to other comparator medications so the resulting profiles could be compared indirectly. In addition, these methods could be applied not only to assess the benefit‐risk profile of a given medication for a specific patient population but also to assess appropriateness for an individual patient. In this regard, the benefit‐risk profile of a medication may be acceptable for some patients but less so for others.

## Conclusions

Use of a multi‐method approach incorporating both quantitative and qualitative analyses provided a useful way to evaluate the benefit‐risk balance for this medication designed for long‐term use. Based on the frequency analysis under the BRAT framework, benefits of olanzapine LAI such as remission days and relapse‐free days appeared to outweigh lower‐probability events such as PDSS, but higher‐probability risks such as weight gain remained a significant clinical concern for many patients treated for up to two years. Based on the TURBO method, olanzapine LAI's benefit‐risk balance was within the acceptable range.

## Declaration of interest statement

Dr Lauriello has received grants or honoraria from Sunovion and Otsuka and has served on advisory or data monitoring boards for Astra Zeneca, Janssen, Lilly, Otsuka, Shire, and Sunovion; Dr Detke, Dr McDonnell, and Mr Landry are employees and stockholders of Eli Lilly and Company.
